# The Pathogenesis of Saffold Virus in AG129 Mice and the Effects of Its Truncated L Protein in the Central Nervous System

**DOI:** 10.3390/v8020024

**Published:** 2016-02-18

**Authors:** Shawn Zheng Kai Tan, Kaw Bing Chua, Yishi Xu, Mookkan Prabakaran

**Affiliations:** Temasek Life Science Laboratory, 1 Research Link, National University of Singapore, Singapore 117604, Singapore; shawntanzk@tll.org.sg (S.Z.K.T.); chuakb@tll.org.sg (K.B.C.); yishi@tll.org.sg (Y.X.)

**Keywords:** Saffold virus, L protein, central nervous system

## Abstract

Saffold Virus (SAFV) is a human cardiovirus that has been suggested to cause severe infection of the central nervous system (CNS). Compared to a similar virus, Theiler’s murine encephalomyelitis virus (TMEV), SAFV has a truncated Leader (L) protein, a protein essential in the establishment of persistent CNS infections. In this study, we generated a chimeric SAFV by replacing the L protein of SAFV with that of TMEV. We then compared the replication in cell cultures and pathogenesis in a mouse model. We showed that both SAFV and chimeric SAFV are able to infect Vero and Neuro2a cells well, but only chimeric SAFV was able to infect RAW264.7. We then showed that mice lacking IFN-α/β and IFN-γ receptors provide a good animal model for SAFV infection, and further identified the locality of the infection to the ventral horn of the spine and several locations in the brain. Lastly, we showed that neither SAFV nor chimeric SAFV causes persistence in this model. Overall, our results provide a strong basis on which the mechanisms underlying Saffold virus induced neuropathogenesis can be further studied and, hence, facilitating new information about its pathogenesis.

## 1. Introduction

Members of the *cardiovirus* genus of the Picornaviridae are single-stranded RNA viruses thought to mainly infect rodents. However, in 2007, a novel human cardiovirus, designated Saffold virus (SAFV), was identified from the stool sample of a child with a fever of unknown origin [1]. Phylogenetic analysis revealed that SAFV is closely related to the Theilovirus species, which consists of Theiler’s murine encephalomyelitis virus (TMEV), Theiler’s rat virus (TRV), and Vilyuisk human encephalomyelitis virus (VHEV) [2]. Since then, SAFVs have been isolated from nasal and stool specimens from children with respiratory and gastrointestinal symptoms in various parts of the world [1,3,4,5,6,7,8]. The SAFV genome is approximately 8050 nucleotides that encodes for the leader (L) protein, four capsid proteins (VP1 to VP4), and seven non-structural proteins (2A, 2B, 2C, 3A, 3B, 3C, and 3D) [1]. To date, 11 genotypes of SAFV have been identified based on phylogenetic analysis of the VP1 gene [9]. While SAFV-2 and SAFV-3 are highly prevalent in humans [5], the pathogenicity of this virus still remains unclear.

Previously, Hertzler, *et al.* [10] found that intracerebral inoculation of SAFV-2 to FVB/n (an inbred mouse strain commonly used for nonclinical drug discovery) mice causes neuropathological changes consistent with acute encephalomyelitis. While the study above has started to uncover the pathogenesis of SAFV, there is a lot more yet unknown. Furthermore, as reviewed by Himeda and Ohara [9], there is a need for an established animal model of SAFV infection and more studies to clarify its virulence potential and its impact on global health.

TMEV, a virus that is structurally and functionally similar with SAFV [11], has been extensively studied due to its ability to cause persistent infections [9]. TMEV is divided into two subgroups based on their neurovirulence after intracerebral inoculation [12]; the GDVII strain causes acute fatal encephalitis, killing all infected mice within two weeks, while DA strain causes milder encephalomyelitis, which then progresses to persistent infection and progressive demyelination reminiscent of multiple sclerosis [9].

The leader (L) protein located at the N-terminal portion of the polyprotein of TMEV have been shown to have an essential role in the differences seen between the two TMEV groups [13]. The typical characteristics of the L region of TMEV, and other similar cardioviruses, contain a well-conserved zinc-finger motif, an acidic region and a serine/threonine-rich domain [14]. Interestingly though, the serine/threonine-rich domain of L protein is partially deleted in SAFV [5]. When analyzed, the homology of L between SAFV and TMEV DA strains is 78%. While a chimeric SAFV containing TMEV L protein has been previously developed and studied [15], the authors only explored its role in suppression of interferon, while the functional impact and difference in specific biological activities caused by this difference is still relatively unknown.

In this study, we had three objectives. Firstly, we wanted to establish AG129 mice (mice that have an intact immune system, but lacks alpha/beta interferon (IFN-α/β) and IFN-γ receptors [16]) as an animal model for SAFV infection. Secondly, we wanted to study the neuropathogenesis of SAFV. Lastly, we wanted to question if the L protein of SAFV is functionally similar to that of the TMEV DA strain, or if the differences in the sequences of L causes any specific differences in biological activities. In this study, we used SAFV-3 due to the high prevelance of infection in nature [5]. In order to achieve our objectives, we generated chimeric SAFV from a full-length infectious cDNA clone, replacing the gene coding for L protein with that of the DA strain of TMEV. We then observed the differences in virus replication and neuropathogenesis of chimeric SAFV and SAFV in a mouse model.

## 2. Materials and Methods

### 2.1. Cell Lines and Virus

Vero (CCL-81), mouse neuroblastoma (Neuro2A, CCL-131), and mouse macrophages (RAW264.7, TIB-71) cells were previously obtained from American Type Culture Collection. All cells were grown in Dulbecco’s modified Eagle’s medium (DMEM, Gibco, Grand Island, NY, USA) containing 10% fetal bovine serum (FBS) and penicillin-streptomycin at 37 °C in 5% CO_2_. The SAFV (Penang strain) was previously isolated from a throat swab specimen obtained from a five-year-old girl with acute influenza-like illness in Penang, Malaysia, in 2009 [17] and classified as genotype 3 (HQ162476.1) based on the VP1 gene sequence.

### 2.2. Construction of Infectious cDNA Clone of Saffold Virus

**Figure 1:**
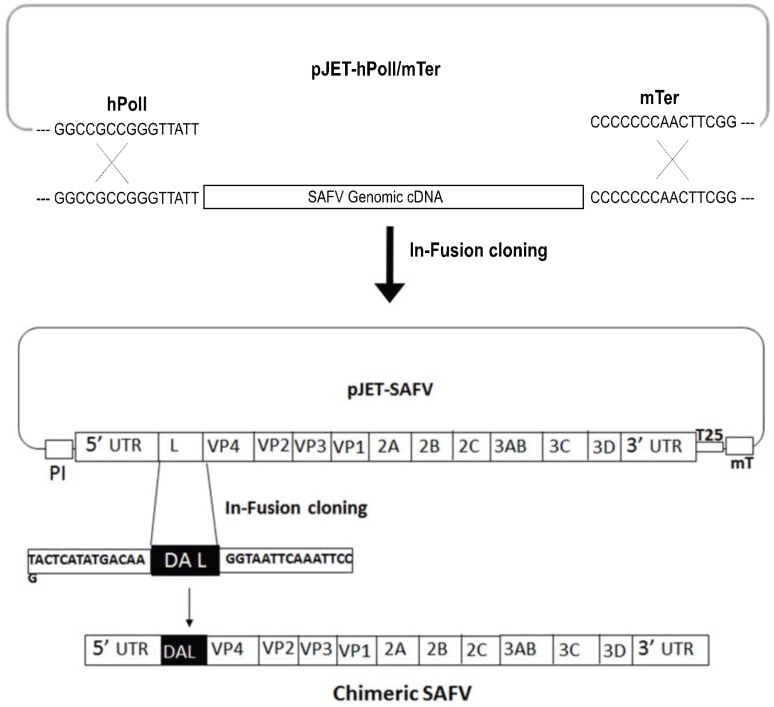
Strategy for the construction of SAFV and chimeric SAFV constructs. The pJET-SAFV plasmid was generated by insertion of SAFV cDNA amplicon into pJET-hPolI/mTer. Further, DA L gene from TMEV was flanked with linear pJET-SAFV between 5'UTR and VP4 using an In-Fusion cloning method. After transfection of pJET-DAL-SAFV into Vero cells, chimeric SAFV virus was rescued. PI: human polymerase I promoter; 25: poly (A) tail with 25 As; mT: murine terminator.

Viral RNA was extracted from SAFV-Penang in Vero cells using TRIzol reagent (Invitrogen, Gaithersburg, MD, USA). Then, RNA was reverse-transcribed by a RevertAid First Strand cDNA synthesis kit (Fermentas, Vilnius, Lithuania) using oligo (dT) 18 as a primer. The full-length SAFV genomic cDNAs were amplified by the PCR using an iProof High-Fidelity GC Master Mix (Biorad Laboratories GmbH, Munich, Germany) with the following primer set; forward primer, SAFV-F (5'-GGCCGCCGGGTTATTTTTCAAAGGGGGCCCTGGGGTC) and reverse primer, SAFV-R (5'-CCGAAGTTGGGGGGGTTTTTTTTTTTTTTTTTTTTTTTTTTTGTTCTCATTTCCAATTAAAAGCT). The reverse primer includes the sequences of the 3' end of SAFV and murine terminator sequences. The pJET-hpol1/mTer plasmid was previously generated by insertion of human RNA polymerase I (hpol1) promoter and a murine terminator cassette sequence [18,19]. The plasmid pJET-hPolI/mTer was linearized by PCR using primers pJET-HPol1mTer-f (5'-CCCCCCCAACTTCGGAGGTC) and pJET-hPol1mTer-r (5'-AATAACCCGGCGGCCCAAAATG). Then, purified SAFV cDNA amplicons were cloned into linearized pJET-hPolI/mTer using an In-Fusion HD cloning kit (Agilent Technologies Inc., Santa Clara, CA, USA) without any enzyme digestion (Figure 1). The positive SAFV construct was confirmed by sequencing and was called pJET-SAFV.

### 2.3. Construction of Chimeric Virus

Chimeric cDNA clone was constructed by replacing the L gene of SAFV with the L gene of the DA strain of TMEV (Figure 1). Briefly, the L-coding region of SAFV was removed from the plasmid pJET-SAFV and linearized by PCR using primers pJet-L-del-F (5'-GAGCCCCAGGGTAATTCAAATTCCTCAGATAAG) and pJet-L-del-R (5'-CTTGTCATATGAGTAAAAAAGGAAAACC). Synthetic L gene of Daniels (DA) strain of TMEV (Genescript, Piscataway, NJ, USA) was amplified using primers DA-L-HD-F (5'-TACTCATATGACAAGATGGCTTGTAAACACGGA) and DA-L-HD-R (5'-GGAATTTGAATTACCCTGGGGCTCCATCACAATGTC), bearing the 15-bp homology necessary for InFusion HD cloning, with linearized pJet-Ldel-SAFV (In-Fusion HD cloning kit, Agilent Technologies, Inc.) (Figure 1). The positive chimeric SAFV construct was confirmed by sequencing, and was called pJET-DAL-SAFV.

### 2.4. Transfection and Infection

Sub-confluent Vero cells in six-well plates were transfected with 2 μg of recombinant plasmid pJET-SAFV or pJET-DAL-SAFV using Lipofectamine 2000 reagent (Invitrogen) according to the manufacturer's instructions. Three days post-transfection, cells were lysed by three freeze and thaw cycles, and the supernatant containing viruses was collected for further passage. The rescued viruses of SAFV and chimeric SAFV were passaged on Vero cells, and virus titer was determined by 50% tissue culture infective dose (TCID_50_) (Reed and Muench [20] method). Further, viruses were sequenced to confirm the absence of unwanted mutations.

### 2.5. Anti-SAFV VP1 Polyclonal Antibody Production

The rabbit anti-VP1 polyclonal antibody was produced in the laboratory. Briefly, the VP1 gene was amplified from SAFV cDNA and cloned into pET-28a vector (Novagen, Darmstadt, Germany) followed by transformation into *Escherichia coli* BL21-competent cells for VP1 protein expression. Hexa-histidine-tagged fusion VP1 expression was induced by the addition of 1 mM IPTG overnight and purified on a Ni-NTA column (Qiagen, Valencia, CA, USA). The purified VP1 protein was separately mixed with complete Freund’s adjuvant in a 1:1 ratio and injected into two female New Zealand rabbits (0.5 mg/rabbit). The rabbits were subsequently injected three more times with the protein mixed with incomplete Freund’s adjuvant at two weekly intervals (0.3 mg/rabbit). Rabbit antisera were collected 10 days after the final injection and tested against the purified proteins and SAFV-infected Vero cell lysate by Western blots and Vero cells infected by SAFV by immunofluorescence.

### 2.6. Indirect Immunofluorescence Assay

SAFV or chimeric SAFV infected cells were analyzed by immunofluorescence staining using VP1 specific polyclonal antibodies. Briefly, Vero, RAW 264.7, or Neuro2A cells were infected with SAFV or chimeric SAFV for 48 h at 37 °C with 5% CO_2_. After fixation, cells were permeated with 0.1% Triton X-100 and incubated with rabbit anti-VP1 polyclonal antibody for 1 h at 37 °C. The cells were then incubated with FITC-conjugated swine anti-rabbit antibody (DAKO Cytomation, Copenhagen, Denmark) and counterstained with DAPI (Invitrogen). The fluorescence signal was detected with an inverted fluorescence microscope (Olympus, Essex, UK) and the images were captured by a digital imaging system (Nikon, Tokyo, Japan).

### 2.7. Kinetics of Virus Growth

Growth kinetics of SAFV or chimeric SAFV were assessed as previously described. Briefly, sub-confluent monolayers in 35-mm diameter wells of Vero, RAW 264.7, and Neuro2a cells were infected under multi-step growth conditions with SAFV or chimeric SAFV at a multiplicity of infection (MOI) of 1 for 1 h at 37 °C. Cells were washed twice with phosphate buffered saline (PBS) and 1 mL of medium containing 2% FBS was added. The culture plates were incubated at 37 °C for 8 h, 16 h, 24 h, 48 h, and 72 h. At these time points, cells were lysed by three freeze and thaw cycles and the supernatant containing viruses were stored at −80 °C. Virus titration was performed in triplicate on Vero cells with each cell type and time point replicated three times. The viral titers were calculated and expressed as 50% tissue culture-infectious doses per volume (TCID_50_/mL) using the Reed and Muench method [20].

### 2.8. Ethics Statement

All animal experiments were reviewed and approved by the Institutional Animal Care and Use Committee (IACUC) of the Temasek Life Sciences Laboratory, Singapore (IACUC approval number TLL-14-025). Mice were housed in specific pathogen-free conditions with water and standard food available *ad libtum*, and they were monitored daily for health and condition. All mice were humanely euthanized with CO_2_ inhalation for five minutes when clinical signs of infection become severe (lack of mobility) or if body weight dropped below 80% of baseline weights.

### 2.9. Mouse Infection

Specific-pathogen-free BALB/c mice were obtained from *InVivos* Pte Ltd. (Singapore, Singapore), and AG129 mice were obtained from B and K Universal (North Humberside, UK). All mice were bred and housed in individual ventilated cages (Tecniplast Sealsafe, Buguggiate, Italy) at the animal holding unit of the Temasek Life Sciences Laboratory. Two-week-old BALB/c (*n* = 6) or two-week-old AG129 suckling mice (*n* = 12) were inoculated i.p with 5 × 10^5^ TCID_50_/mouse of SAFV or chimeric SAFV. In a separate study, 3–4-week-old AG129 weanling mice (*n* = 12) were also inoculated the same dose of SAFV or chimeric SAFV virus. Mice (*n* = 6) were observed daily to monitor body weight, clinical signs, and survival rates up to 35 day post-infection (dpi).

### 2.10. Quantification of Infectious Virus in CNS

For determination of viral titer in CNS, the spine and brains from three randomly selected AG129 mice were aseptically removed on day six or 35 days post infection, weighed and stored at −80 °C after euthanasia of mice with CO_2_ inhalation for five minutes. Tissue was then homogenized in 1 mL DMEM by using a TissueLyser LT homogenizer (Qiagen). After the homogenates were frozen and thawed three times, ten-fold dilutions of each organ suspension were prepared and titrated on monolayers of Vero cells. The virus titers were obtained by the endpoint titration method determined by observation of the CPE and also confirmed by immunofluorescence assay using a rabbit anti-VP1 polyclonal antibody described above. The infective titer was calculated by the Reed and Muench method [20] and expressed as log10 TCID_50_/mL. Each assay was carried out in triplicate and the limit of virus detection was 1.5 log10 TCID_50_/mL of tissue specimens.

To further confirm quantification data, we carried out qPCR. Briefly, the RNA from the homogenates were extracted using TRIzol reagent (Invitrogen), and viral RNA copy numbers of tissue were determined by SBGR qRT-PCR. Briefly, the total RNA from each sample was reverse transcribed into cDNA using reverse transcriptase AMV reagents (Roche) with oligo (dt) 18 as primer on standard conditions. qPCR was performed on a Rotor-Gene Q (Qiagen) with standard conditions using SYBR Green reagents and fluorescence for detection. SAFV-VP1 qPCR primers were designed using Primer3 software [21], and GAPDH was used as internal control and all VP1 C_T_ values were normalized using their respective GAPDH C_T_ values. To ensure primer efficiency in qPCR reactions, amplification efficiency was accessed as described by Livak and Schmittgen [22]. Plasmid copies were determined using a standard curve obtained from primer efficiency studies, the formula used was x = (y − 29.965) / −2.7474 where plasmid copies = 10^x^ and y = C_T_ value. Primers used for qPCR are as follows: GAPDH-F (5'-ACCACCATGGAGAAGGC-3'), GAPDH-R (5'-GGCATGGACTGTGGTCATGA-3'), SAFV-VP1-F (5'-TCTCTCCGTCTTACCGTCAGTGT-3'), SAFV-VP1-R (5'-ACAGAAGTCTTCCAAAGTCGGCA-3').

### 2.11. Tissue Preparation

At six or 35 dpi, three mice (*n* = 3) were anesthetized intraperitoneally (i.p.) with Ketamine (100 mg/kg)/Xylazine (20 mg/kg) and transcardially perfused with 50 mL PBS followed by 100 mL 4% paraformaldehyde (PFA) in PBS. The mice were then decapitated and the brains and spine were removed and post-fixed in 4% PFA overnight at 4 °C. Brains were then transferred to a solution of 20% sucrose in PBS and stored overnight at 4 °C before freezing over liquid nitrogen and stored at −80 °C. The brains were then embedded in Shandon M-1 Embedding Matrix (Thermo, Kalamazoo, MI, USA) and sectioned through a cryostat (16 μm). Spines were decalcified with 6% trichloroacetic acid (TCA, Sigma, Saint Louis, MO, USA) for five days at 4 °C and embedded in paraffin wax before being sectioned at 5 μm.

### 2.12. Localization of Virus Infection in CNS by Immunohistochemistry

Tissue sections were blocked with 3% skim milk containing 0.1% Triton X-100 for 1 h at room temperature. Prior to blocking, spine sections were deparaffinized using histochoice (Amresco, Fountain Parkway Solon, OH, USA) and rehydrated in sequential ethanol baths, followed by antigen retrieval by pressure-cooking in citrate buffer for 5 min. For localization of viral antigen, tissue sections were incubated with rabbit anti-VP1 primary antibody (1:500) overnight at 4 °C. Tissue sections were then further incubated with secondary antibody Alexa Fluor 488 conjugated goat anti-rabbit IgG (Invitrogen) for 2 h at RT and counterstained with DAPI (Invitrogen) before coverslipping with anti-fade mounting medium (DAKO). The resulting staining was observed under an upright fluorescence microscopy (Zeiss, Jena, Germany).

### 2.13. Statistics

All statistical analysis was done using GraphPad Prism 5.01 [23]. Statistical models used are stated in the various results sections. Growth kinetics were analyzed using two-way repeated-measure ANOVA with Bonferroni *post hoc* analysis. Viral titers were analyzed using unpaired *t*-test. Results were considered significant when *p* < 0.05.

## 3. Results

### 3.1. Construction of cDNA Clones of SAFV and Chimeric SAFV

The full-length SAFV cDNA amplicons were directly cloned with linearized pJET-hPolI/mTer plasmid using the In-Fusion HD cloning system without any enzyme digestion. Similarly, we also constructed a chimeric SAFV from a full-length infectious SAFV cDNA clone in which the L-coding region of SAFV was replaced with that of the DA strain of TMEV. Then, plasmids that were confirmed by sequencing were transfected into Vero cells, and the SAFV and chimeric SAFV were rescued successfully. To assess whether the rescued SAFV or chimeric SAFV were replication competent, Vero cells were infected with wild-type (wt) SAFV, reverse genetics SAFV, or chimeric SAFV virus and one-step growth curves among the viruses were compared. The titers of wt-SAFV virus reached 1.74 × 10^9^ TCID_50_/mL at 48 h post infection (hpi). Similarly, SAFV and chimeric SAFV showed 2.14 × 10^9^ TCID_50_/mL and 1.62 × 10^9^ TCID_50_/mL respectively, at 48 hpi.

### 3.2. Replication of SAFV and Chimeric SAFV in Different Cell Lines

**Figure 2:**
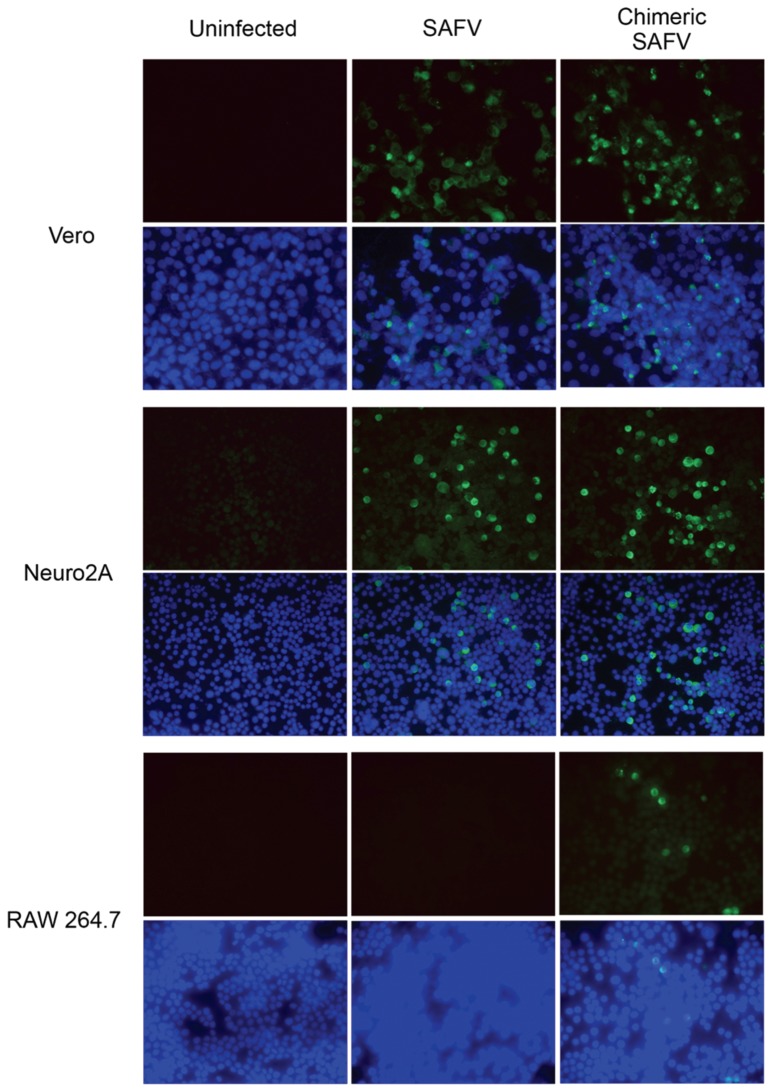
Immuno-fluorescence assay of Vero, Neuro2a, and RAW 264.7 cells infected with SAFV or chimeric SAFV for 48 h at 37 °C with 5% CO_2_. Anti-VP1 signal was seen in both SAFV and chimeric SAFV infected Vero and Neuro2A cells. A faint signal was observed in chimeric SAFV infected RAW 264.7 cells but not SAFV infected RAW 264.7 cells.

Immunofluorescence assay for detection of virus-infected cells demonstrated that SAFV or chimeric SAFV infected both Vero and Neuro2a cells. In contrast, no fluorescent signals were seen for SAFV infected RAW 264.7 cells and very few fluorescent-positive cells were seen for chimeric SAFV infected RAW 264.7 cells (Figure 2).

### 3.3. Growth Kinetics of SAFV and Chimeric SAFV

**Figure 3:**
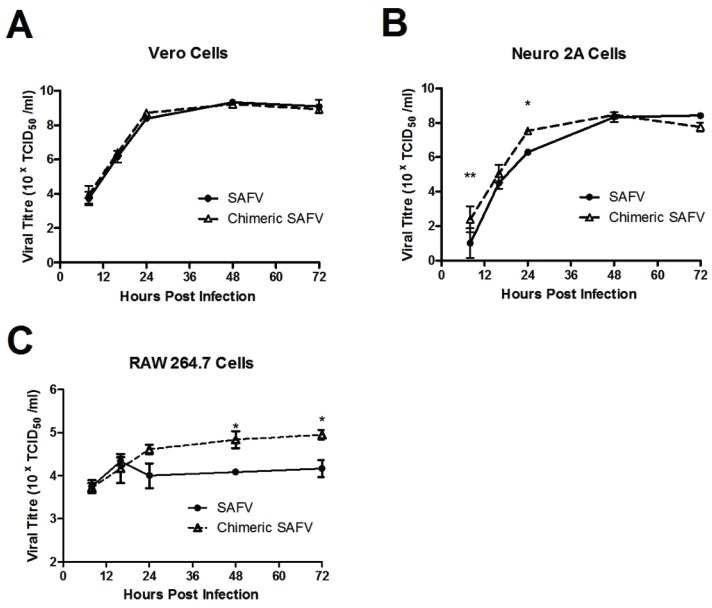
Growth Kinetics of SAFV or chimeric SAFV in Vero (**A**); Neuro2a (**B**) or RAW 264.7 (**C**) cells. Data presented in mean ± SEM. There was an effect in time in all cells, an effect of virus in Neuro2A and RAW 264.7, but not Vero cells, and an effect of interaction only in RAW 264.7, but not Vero or Neuro2A cells. Bonferroni *post hoc* analysis of Vero cells revealed no significance at any time point. Bonferroni *post hoc* analysis of Neuro2A cells revealed significannce difference at eight and 16 h time point. Bonferroni *post hoc* analysis of RAW 264.7 cells revealed difference at 48 and 72 h time point. ** represents *p* < 0.01, * represents *p* < 0.05.

The replication kinetics of SAFV was compared with chimeric SAFV in Vero, Neuro2a, and RAW 264.7 cells. A two-way repeated-measures ANOVA showed an effect in time (smallest F = 5.58, all *p*s < 0.01) in all cells, an effect of virus in Neuro2A (F_(1,4)_ = 11.86, *p* = 0.026) and RAW 264.7 (F_(1,4)_ = 15.44, *p* = 0.017), but not Vero (F_(1,4)_ = 1.36, *p* = 0.31), and an effect of interaction in Neuro2A (F_(4,16)_ = 5.29, *p* = 0.007), but not Vero or RAW 264.7 (biggest F = 2.82, *p*s > 0.05). Bonferroni *post hoc* test revealed a significant difference at eight and 14 h for Neuro2A, and 48 and 72 h for RAW 264.7 (Figure 3).

### 3.4. Pathogenesis of SAFV or Chimeric SAFV

Intraperitoneal infection of AG129 suckling mice with SAFV and chimeric SAFV exhibited greater susceptibility to infection than those of weanling mice. AG129 suckling mice infected with either SAFV or chimeric SAFV showed ruffled fur and hunched posture from four days post infection (dpi) and hind limb paralysis within 6 dpi and all mice either succumbed to death or were humanely euthanized because of severe diseases (two-limb paralysis or inability to move or moribund) by 8 dpi. Administration of either SAFV or chimeric SAFV to BALB/c mice of the same age showed no clinical symptoms up to day 35. I.P. Administration of chimeric SAFV to AG129 weanling mice caused 33.3% to succumbed to severe hind limb paralysis between 8 and 11 dpi during which they were humanly euthanized. Mice that survived the chimeric SAFV infection showed minor clinical symptoms between 9 and 12 dpi and remain healthy throughout an observation period of 35 dpi. Interestingly, AG129 weanling mice administered with SAFV showed minor clinical symptoms between 7 and 10 dpi and then no abnormal clinical features observed up to 35 dpi.

### 3.5. Viral Titers in CNS

**Figure 4:**
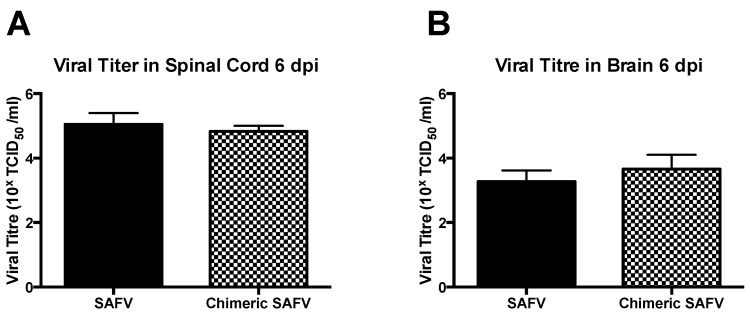
Virus titers in CNS 6 dpi. Data presented in mean + SEM. Two-week-old AG129 was infected with either SAFV or Chimeric SAFV, spine and brain were obtained 6 dpi, and viral titer was determined by TCID_50_ using Reed and Muench method, Unpaired *t*-test revealed no significant difference seen between viral titers of SAFV and Chimeric SAFV in both spine (**A**) and brain (**B**).

Suckling AG129 mice administered with either SAFV or chimeric SAFV showed 10^5.05 ± 0.20^ TCID_50_/mL and 10^4.83 ± 0.10^ TCID_50_/mL, respectively, in spine, and 10^3.27 ± 0.20^ TCID_50_/mL and 10^3.66 ± 0.25^ TCID_50_/mL, respectively, in brain on 6 dpi (choosen based as a predetermined endpoint based on clinical symptoms mentioned above). An unpaired *t*-test showed no significant differences between viruses in both spine (t_(4)_ = 0.98, *p* = 0.38), and brain (t_(4)_ = 1.21, *p* = 0.29) (Figure 4). BALB/c mice showed no detectable titer in both spine and brain. Weanling mice infected with either SAFV or chimeric SAFV showed no detectable viral titer on 35 dpi.

qPCR analysis of viral copies/μg RNA revealed no significant difference between SAFV and chimeric SAFV in both spine and brain. An unpaired *t*-test with Welsch correction revealed no significant difference between SAFV and chimeric SAFV in both spine (T_(7.82)_ = 1.01, *p* = 0.34) and brain (T_(7.19)_ = 0.88, *p* = 0.41). Using standard curve comparison, the average viral copies/μg RNA are as follows: SAFV Spine = 8.81 × 10^5^, Chimeric SAFV Spine = 4.49 × 10^5^, SAFV Brain = 3.96 × 10^5^, and Chimeric SAFV Brain = 3.51 × 10^5^.

### 3.6. Immunohistochemical Detection of Viral Antigen in Spine Sections

**Figure 5:**
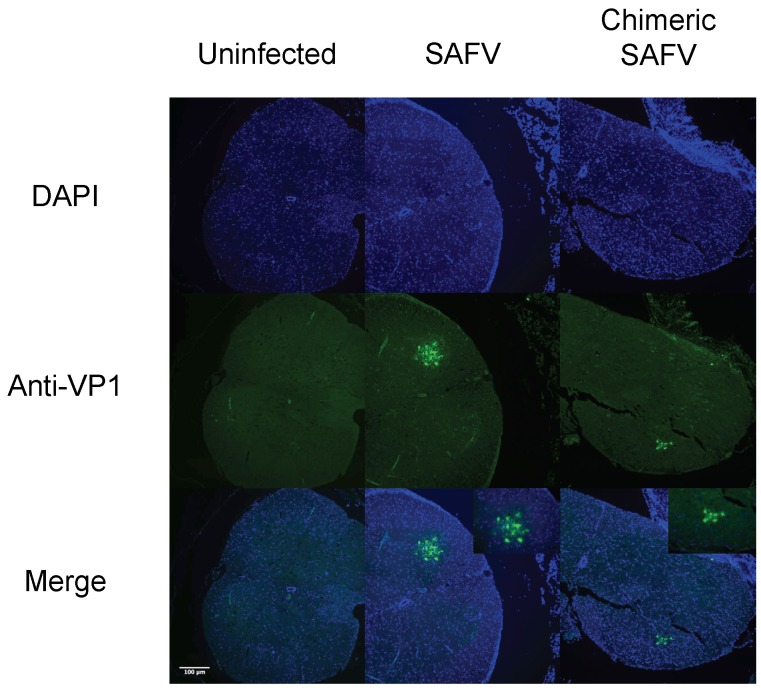
Fluorescence images of uninfected, SAFV infected, and chimeric SAFV infected spine sections. Two-week-old AG129 was infected with either SAFV or Chimeric SAFV, and spine was obtained 6 dpi. Spine sections were stained with anti-VP1 antibodies (Green) and counterstained with DAPI (Blue). SAFV and chimeric SAFV infected sections show staining at the ventral horn.

To observe the spread of virus in the spine of AG129 mice on 6 or 35 dpi, sections of infected mice spine underwent immunohistochemistry using a polyclonal antibody against SAFV VP1. Staining was observed at the ventral horn of the gray matter for both SAFV and chimeric SAFV infected mice on 6 dpi (Figure 5). However, no positive staining was detected in the spine of SAFV or chimeric SAFV infected weanling mice that survived on 35 dpi (Figure S1).

### 3.7. Immunohistochemical Detection of Viral Antigen in Brain Sections

**Figure 6:**
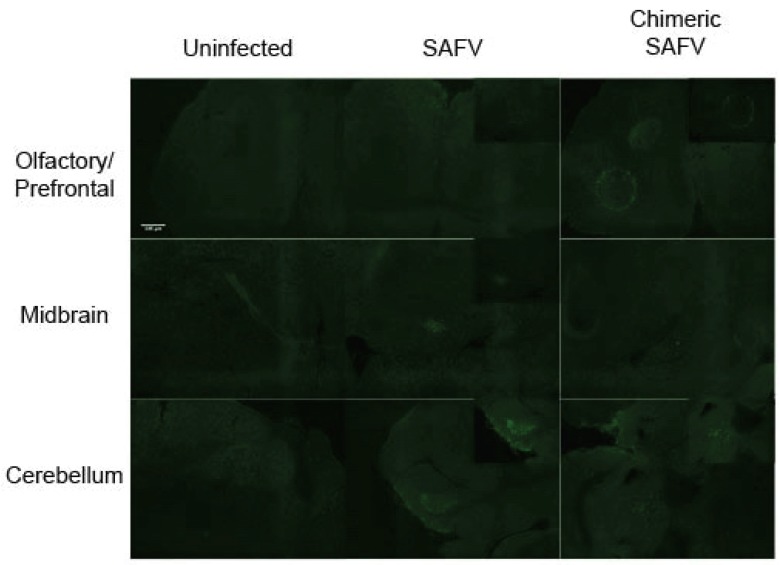
Fluorescence images of uninfected, SAFV infected, and chimeric SAFV infected brain sections. Two-week-old AG129 was infected with either SAFV or Chimeric SAFV, and brain was obtained 6 dpi. Sections were stained with anti-VP1 antibodies (Green). SAFV infected brains show staining in the olfactory/prefrontal area, midbrain, and cerebellum. Chimeric SAFV infected brains show staining in the olfactory/prefrontal area, and cerebellum, but not in the midbrain.

To observe the spread of the virus in the brain, sections of infected mice brains underwent immunohistochemistry using a polyclonal antibody against VP1. Staining was observed at the olfactory bulb, midbrain area, and cerebellum for both SAFV and chimeric SAFV infected mice on 6 dpi (Figure 6). In contrast, viral antigen was not detected in brain sections of mice that survived from either SAFV or chimeric SAFV infection on 35 dpi (Figure S1).

## 4. Discussion

Since its discovery in 2007, 11 genotypes of Saffold Virus have been identified and it has been found to be widespread and causes infection early in life in humans [5,24]. Despite this, very little is known about the pathogenicity of this virus. Previous reports have, however, suggested that SAFV may cause severe infections of the central nervous system. Furthermore, SAFV has been isolated from the stool sample of a patient with non-polio acute flaccid paralysis [9]. TMEV is an extensively studied virus that is structurally and functionally similar with SAFV [11] and, hence, presents a good method to further understand the replication and pathogenicity of SAFV. Reverse-genetics is a powerful tool for the study of viral replication and pathogenicity. A previous study has generated infectious RNA *in vitro* from full length cDNA of SAFV using T7 RNA polymerase [25]. The main limitation is that this method is laborious and any mutations introduced by proof-reading deficient T7 RNA polymerases may affect viability of infectious particles. In this study, we generated infectious cDNA clone of SAFV under the control of a hRNA pol 1 promoter. After transfection into Vero cells, the genomic viral cDNA was transcribed into SAFV genomic RNA by hpol1 inside the cells. This method eliminates the need for troublesome *in vitro* RNA transcription and the need to addition extra bases during *in vivo* transcription at the 5' and 3' ends of viral transcripts [19].

The L protein of TMEV has been shown to play an essential role in the establishment of persistent CNS infections in mice [13]. This sequence is, however, partially deleted in SAFV. To understand the effect of this partial deletion on neurovirulence, we created a chimeric virus by replacing the sequence coding for SAFV L protein with that of the L protein of TMEV DA strain. This is not the first study to have generated and studied a chimeric SAFV with L protein of TMEV. Recently, Shimizu, *et al.* [15] similarly generated a chimeric SAFV by replacing the L protein of SAFV with that of TMEV DA strain. While they did study the growth kinetics of TMEV DA strain with SAFV L in the J774 macrophage cell line, they did not report any findings on growth kinetics of SAFV with TMEV DA L. In this paper, we examined the replication of SAFV and chimeric SAFV in multiple cell lines, and developed a mouse model to study neuropathological changes following infection with the viruses.

In order to observe if there were any differences in infectivity between SAFV and chimeric SAFV, we infected Vero, Neuro2a, and RAW 264.7 cell lines. We showed that SAFV was able to infect Vero and Neuro2a cells well, but not RAW 264.7 cells. This replicates results from Xu, *et al.* [26] which also showed SAFV infectivity in Vero and Neuro2A, but not RAW 264.7. Chimeric SAFV replicated more effectively compared to SAFV early on in Neuro2A cells, which suggests that the L protein of TMEV might be more active than that of SAFV. Interestingly, chimeric SAFV was also able to infect RAW 264.7 cells, though not well. Cell replication kinetics show higher viral levels in later time points in chimeric SAFV compared to SAFV. This is significant as previous studies done with TMEV has shown effective infection of macrophage cell lines, such as RAW 264.7 [27] and J774 [28], and that L* (a small out-of-frame protein coded by an alternative translation initiation site) is important for growth in macrophage cell lines [29,30]. SAFV lacks the AUG initiation codon required to translate L* protein, though it is unclear if the ACG present in that region of SAFV is used to produce a truncated L* protein [9]. This lack or truncation of L* protein in SAFV could explain why SAFV is unable to infect RAW 264.7 cells while chimeric SAFV (which contains TMEV DA L and, hence, would be able to produce L* protein) is able to. This is important as virus persistence in monocytes/macrophages is essential in TMEV induced demyelination [31,32]. The low infectivity of RAW 264.7 cells by the chimeric SAFV however do suggest that more factors than L* are required for proper infection. The structural capsid proteins of TMEV, which have been shown to be important for receptor binding [33], are completely different from that of TMEV. Taken together, these results suggest that the capsid proteins of TMEV are also important in macrophage infection and persistence

Initially, we tried infecting BALB/c mice to test if it was an appropriate model to use for these viruses. We, however, found neither clinical symptoms nor detectable viral titer in the central nervous system after i.p. infection with SAFV or chimeric SAFV, making it an inappropriate model for the viruses (data not shown). AG129 mice have an intact immune system, but lacks alpha/beta interferon (IFN-α/β) and IFN-γ receptors [16], supporting the spread and persistence of viruses. Interferons have been shown to be important in the resistance/susceptibility in TMEV [34]. Previous studies revealed that inbred 129Sv mice lacking IFN-α/β receptors showed severe encephalomyelitis and died (acute TMEV infection) whereas mice lacking IFN-γ receptors were highly susceptible to persistent infection in the white matter of the brain and spine [34,35]. We reasoned that a mouse model with both IFN-α/β and IFN-γ knocked out would be appropriate as we wanted to stop both rapid responses by IFN-α/β, hence allowing the early spread of infection, and also late stage immune-mediated responses, and IFN-γ, hence allowing persistence. Furthermore, AG129 has previous been shown to allow neurological infection of other non-mouse-adapted viruses [36,37]. Overall, AG129 mice seemed to be permissive to SAFV infection and hence a good model to use for these viruses.

In our study, we found that i.p. infection of SAFV and chimeric SAFV in suckling AG129 mice causes ruffled fur, hunched posture and, subsequently, hind-limb paralysis and death. These results are similar to the study by Hertzler, *et al.* [10] in which they inoculated SAFV-2 intracerebrally to six-week-old (adolescent) FVB/n mice and found similar symptoms. When immunohistochemical staining was done using VP1 antibodies, we found virus present in the ventral horn of the spine grey matter, olfactory bulb/prefrontal area of the brain, midbrain, and cerebellum of infected mice. Previous animal work with SAFV has suggested the virus is neurotropic in mice [9,38] however, to our knowledge, this is the first paper to determine the locality of SAFV in the CNS.

Infection in the spine was localized to the ventral horn (which contains motor neurons), which could potentially be the site in which the virus causes paralysis. Further studies are, however, required to determine the mechanism in which the virus causes paralysis. Infection in the brain was found mainly at the olfactory bulb/prefrontal area and the cerebellum, with SAFV showing infection in the midbrain area (but not chimeric SAFV). It is unclear as to how the virus travels to the brain and the reasons and mechanisms behind the areas they infect. Furthermore, due to the paralysis and the rapid nature of infection, we were unable to determine if there were any behavioral changes caused by brain infection. Given the specificity of locality in the brain, we do believe that this data warrants further investigation. While we did manage to elucidate the locality of the virus in general structures of the CNS, we were unable to determine cellular and subcellular locality of the virus. Further work looking into the cellular locality of the virus could help further elucidate the mechanisms in which this virus causes paralysis.

Weanling mice infected with SAFV only showed minor symptoms and subsequently managed to recover from it. This reflects humans as well as, to our knowledge, SAFV has only been detected in young children [1,3,4,5,7,8,11,17]. When analyzed 35 dpi, mice showed no symptoms (after initial symptoms subsided), nor viral signal. Interestingly, weanling mice infected with chimeric SAFV showed susceptibility to infection and manage to kill 33% of mice. Mice that survived did not show any further symptoms, nor viral signal 35 dpi. A possible reason why chimeric SAFV did not cause persistent infection, despite having TMEV DA L, could be a result of it not being able to infect macrophages well; persistent TMEV DA infection is thought to be a result of virus using circulating macrophages that cross the blood-brain barrier as host [39]. However further studies are required to understand this, including studying the role of the capsid protein VP1 and VP2 (particularly CD loop of VP1 and EF loop of VP2), which has been found to be important in persistence (at least in TMEV) [40,41,42]. Regardless, taken together with the clinical study by Galama, *et al.* [43], which suggested that an association between SAFV and multiple sclerosis (a demyelinating disease) is highly improbable, it is highly improbable that SAFV causes persistent infection.

## 5. Conculsions

In conclusion, we showed that AG129 mice (that lack IFN-α/β and IFN-γ receptors) are a good animal model for studying SAFV infections and we identified the locality of infection in the CNS. We further showed that the truncation of L protein, or perhaps lack of or truncation of L*, in SAFV is important for its inability to infect macrophage cells (at least RAW 264.7 cells) and lowered rate of infection (compared to chimeric SAFV). Our results provide a strong basis on which the mechanisms underlying Saffold virus-induced neuropathogenesis can be further studied and, hence, facilitating new information about its pathogenesis. Much more work is required, however, before we can fully understand the pathogenesis of SAFV and the role L protein plays in it.

## Supplementary Materials

The following is available online at www.mdpi.com/1999-4915/8/1/24/s1. Figure S1: Fluorescence images of spine and brain sections of 35 dpi mice.
