# Supplementary material for: The Pathogenesis of Saffold Virus in AG129 Mice and the Effects of Its Truncated L Protein in the Central Nervous System
Source: Viruses. 2016 Feb 18;8(2):24. doi: 10.3390/v8020024 (PMC4776182; doi:10.3390/v8020024)
Supplement: Supplementary file 1 [file viruses-08-00024-s001.pdf]

Supplementary Materials: The Pathogenesis of Saffold Virus in AG129 Mice and the Effects of Its Truncated L Protein in the Central Nervous System

Shawn Zheng Kai Tan, Kaw Bing Chua, Yishi Xu and Mookkan Prabakaran

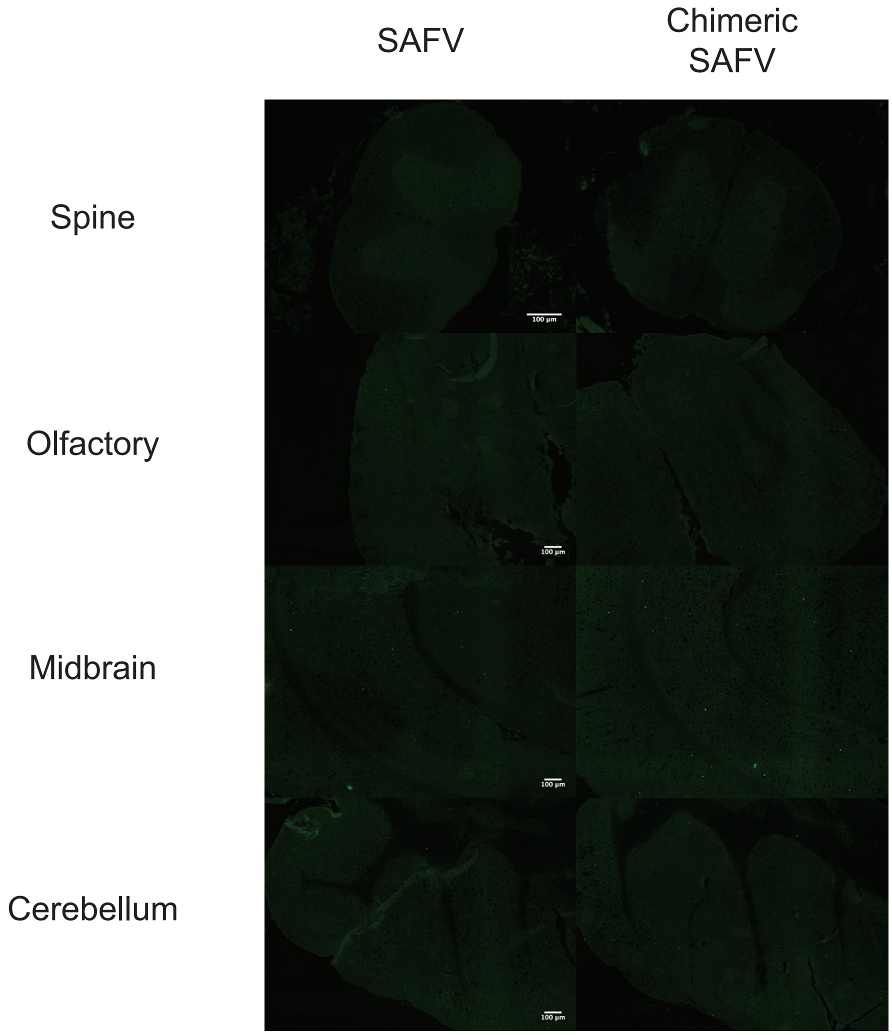

Figure S1. Fluorescence images of spine and brain sections of 35 dpi mice that survived infection, stained with anti-VP1 antibodies. No observable staining was detected.

© 2016 by the authors; licensee MDPI, Basel, Switzerland. This article is an open access article distributed under the terms and conditions of the Creative Commons by Attribution (CC-BY) license (http://creativecommons.org/licenses/by/4.0/).
